# PW02-040 - Low-penetrance NLRP3 variants

**DOI:** 10.1186/1546-0096-11-S1-A181

**Published:** 2013-11-08

**Authors:** T Endres, F Hofer, R Goldbach-Mansky, HM Hoffman, N Blank, K Krause, C Rietschel, G Horneff, P Lohse, J Kuemmerle-Deschner

**Affiliations:** 1 Department of Pediatrics, Division of Pediatric Rheumatology, University Hospital Tuebingen, Tuebingen, Germany; 2 Translational Autoinflammatory Disease Section, NIAMS/NIH, Bethesda, MD, USA; 3 Division of Rheumatology and Allergy/Immunology, University of California at San Diego, San Diego, CA, USA; 4 Hämatologie, Onkologie u. Rheumatologie, Universitätsklinikum Heidelberg, Heidelberg, Germany; 5 „Allergie-Centrum Charité“, Klinik für Dermatologie, Charité Campus Mitte, Berlin, Germany; 6 Kinder- und Jugendrheumatologie, Clementine-Kinderhospital, Frankfurt, Germany; 7 Abteilung für Allgemeine Kinder- und Jugendmedizin, Asklepios-Klinik Sankt Augustin, Sankt Augustin, Germany; 8 Institut für Laboratoriumsmedizin und Humangenetik, Singen, Germany

## Introduction

Cryopyrin-associated periodic syndrome (CAPS) presents as rare, autosomal dominant disease spectrum, due to mutations in the *NLRP3* gene which result in an excessive interleukin-1 (IL-1) release.

In patients with low-penetrance NLRP3 variants, the clinical presentation varies widely. So far, a correlation with a specific phenotype could not be demonstrated.

## Objectives

The aim of this study was to analyze the association of the V198M, R488K, and Q703K substitutions with a specific phenotype, laboratory markers, and the response to IL-1 inhibitors anakinra and canakinumab.

## Methods

This multi-center observational study included 44 patients (25 children and 19 adults). All patients were symptomatic with some symptoms suggesting possible CAPS at the time of baseline examination. Genetic analysis detected one of the following NLRP3 variants: Q703K (n=18), R488K (n=6), and V198M (n=20).

Clinical phenotypes were described and laboratory markers were analyzed. In order to review the response to IL-1 inhibitors, data from follow-up visits were also evaluated.

## Results

At baseline examination, patients reported signs of systemic inflammation such as fever (75%), headache (73%), musculoskeletal symptoms (84%), and fatigue (77%). Other CAPS-specific features were rash (82%), conjunctivitis (43%), and sensorineural hearing loss (25%).

More than half of the patients (57%) reported abdominal pain and other gastrointestinal symptoms. A history of gastro-esophageal reflux was described by 23% of the patients, and 39% of the patients had oral ulcers.

Inflammation markers were only slightly increased: ESR was elevated in 26% (n=34) and C-reactive protein (CRP) in 38% (n=40).

Serum amyloid A (SAA) was raised in 36% (8/22) of the patients. Eight out of nine patients (89%) had elevated TNF-α-levels at baseline examination.

At baseline evaluation, 25 patients were treated with IL-1 inhibitors (anakinra or canakinumab). Data from follow-up visits during the first year of treatment were available from 21 patients: clinical disease activity was reduced in all cases; five patients (24%) achieved full remission, 13 (62%) still had mild symptoms, and three patients (14%) showed only a partial response.

## Conclusion

Heterozygous carriers of NLRP3 variants V198M, R488K, and Q703K display distinct clinical characteristics compared to CAPS patients with definite disease causing mutations, including a high incidence of gastrointestinal symptoms, only slightly elevated inflammatory parameters, and a potentially inferior response to IL-1 inhibition.

## Competing interests

None declared

